# Roundup^®^, but Not Roundup-Ready^®^ Corn, Increases Mortality of *Drosophila melanogaster*

**DOI:** 10.3390/toxics7030038

**Published:** 2019-07-31

**Authors:** Becky Talyn, Rachael Lemon, Maryam Badoella, Darwin Melchiorre, Maryori Villalobos, Raquel Elias, Kelly Muller, Maggie Santos, Erik Melchiorre

**Affiliations:** 1 College of Natural Science, California State University, 5500 University Parkway, San Bernardino, CA 92407, USA; 2 Biology Department, California State University, 5500 University Parkway, San Bernardino, CA 92407, USA; 3 Canyon Kids Academy, 7883 Stewart Road, Colton, CA 92324, USA; 4 Chemistry and Biochemistry Department, California State University, 5500 University Parkway, San Bernardino, CA 92407, USA; 5 Geology Department, California State University, 5500 University Parkway, San Bernardino, CA 92407, USA

**Keywords:** Roundup^®^, glyphosate, LC_50_, *Drosophila melanogaster*, herbicide tolerant, genetically modified food

## Abstract

Genetically modified foods have become pervasive in diets of people living in the US. By far the most common genetically modified foods either tolerate herbicide application (HT) or produce endogenous insecticide (Bt). To determine whether these toxicological effects result from genetic modification *per se*, or from the increase in herbicide or insecticide residues present on the food, we exposed fruit flies, *Drosophila melanogaster*, to food containing HT corn that had been sprayed with the glyphosate-based herbicide Roundup^®^, HT corn that had not been sprayed with Roundup^®^, or Roundup^®^ in a variety of known glyphosate concentrations and formulations. While neither lifespan nor reproductive behaviors were affected by HT corn, addition of Roundup^®^ increased mortality with an LC_50_ of 7.1 g/L for males and 11.4 g/L for females after 2 days of exposure. Given the many genetic tools available, *Drosophila* are an excellent model system for future studies about genetic and biochemical mechanisms of glyphosate toxicity.

## 1. Introduction

The use of genetically modified crops (GMOs) has increased dramatically in industrial agriculture since marketing began in 1996, first in herbicide tolerant (HT) soybeans and insect tolerant (Bt) corn, potato, and cotton. By 1998 Ht cotton and canola were also available. The HT trait allows crops to be sprayed with Roundup^®^ or other glyphosate-based herbicides during the growing season, while Bt crops produce insecticide within the plant tissues. These are still the two most common traits in commercially available GMO crops, including many varieties of corn grown commercially that are genetically modified to have both traits, often including multiple versions of Bt (stacked). Together, HT and Bt crops accounted for 92% of corn [1], 95% of soybeans [1], 95% of canola [2], and essentially 100% of sugar beets [2] planted for commercial production in the US in 2016 or 2017. Worldwide, they accounted for 78% of soybeans, 33% of corn, and 24% of canola in 2016 [3]. GMO cotton, alfalfa, squash, eggplant, papaya, apples, and potatoes were also grown commercially in 2016 and apples starting in 2017.

GMOs are widely considered safe or controversial (e.g., [4,5,6]), based on a combination of experimental and theoretical grounds. For example, HT crops are considered safe because animals are not the target species of the toxin, the herbicide glyphosate (sold as Roundup^®^ among others), which can be sprayed on HT crops throughout the growing season without killing the crop. Animals should not be affected by the primary mechanism of glyphosate, interference with the shikimate pathway of amino acid synthesis [7]. A recent review of the effects of GMO feed on livestock concluded that there is no clear evidence that GMO feed adversely influences animal health [8]. However, this review ignores reproductive outcomes, and cites but discounts seventeen studies that did illuminate health concerns. Many studies now indicate that exposure to GMOs affects health in lab animals, livestock, and aquatic animals (reviewed in [9,10,11,12]. Animal GMO-feeding studies have identified health concerns affecting multiple organ systems including cardiovascular [13,14], digestive [15,16,17], exocrine [18,19], hepatorenal [9,13,20,21,22,23,24,25], immune [26,27,28], and reproductive systems [15,23,27,29,30,31]. Within these systems, traits that differed between GMO-exposed and control groups include cell proliferation [9,32], histology [15,16,18,23,30,31], hematology [14,20,25,28], metabolism [19,21,23,27,31,33,34], milk production [27], and lifespan and mortality [9,29]. Some of these studies [15,17,23,31] used feed containing both HT and Bt ingredients, while others exposed animals to diets containing either HT [9,13,18,19,21,22,25,27,30,33,34,35] or Bt [14,16,20,24,25,26,28,29,32] ingredients. However, most of these studies do not address the mechanism that does cause harm from GMO exposure. Specifically, it is unclear whether GMOs lead to negative health consequences because of unintended alterations resulting from genetic manipulation itself, or directly or indirectly because of the trait (proteins coded by the genes) that has been artificially inserted. For example, the various CRY proteins that are expressed continuously and ubiquitously in some Bt crops may negatively impact health. Since HT GMO crops are sprayed with Roundup^®^ or other glyphosate-based herbicides during the growing season, and therefore often contain higher concentrations of glyphosate residues [35,36,37,38], the herbicide (glyphosate and/or other ingredients, see [33,34]), rather than the genetic manipulation, could cause the negative effects of a GMO-containing diet on animals.

In 2012, world pesticide use (including herbicides, insecticides, fungicides, miticides, rodenticides, etc.) totaled nearly six billion pounds, of which the US accounted for 20% [39]. Glyphosate is the most commonly used pesticide in the US and worldwide, as the active ingredient of Roundup^®^ and several other herbicide formulations. In 2012, glyphosate accounted for 23% (280 million pounds) of the nearly 1.2 billion pounds of total pesticide used in the US—four times as much as atrazine, the next most commonly used pesticide [39]. It is used extensively by consumers for managing weeds around homes, and by communities in maintaining schools, parks, and other public spaces, collectively accounting for almost 9.6% of 2014 glyphosate use [40]. The remaining 90.4% is used in agriculture, making glyphosate the most widely used agricultural pesticide, increasingly so as corn, soy, canola, and sugar beet industries have become dominated by HT crops; and because glyphosate-based herbicide is used as a pre-harvest desiccant to kill foliage on many kinds of grain and sugar cane to synchronize and simplify harvesting.

This high level of glyphosate use results in the detection of glyphosate residues (as well as the primary metabolite, Aminomethylphosphonic acid (AMPA)) in a variety of foods, including in processed foods at levels up to 1125 ppb [41]. In soybeans, glyphosate concentration depends on agricultural practices, such that herbicide tolerant soybeans contained 3.3 mg glyphosate/kg soybean, while none was detected in organically grown soy [42]. In a review of seven studies, mean glyphosate concentration in human urine ranged from 0.21–3.2 ug/L, and in one of those studies, which focused on farmers and their families, the maximum glyphosate concentration in human urine was 233 ug/L [43]. Two studies published since then found that 57.5% of urine samples collected in 2012 in Germany contained glyphosate at concentrations up to 2.8 ug/L [44]; while in Southern California prevalence of glyphosate in urine increased from 1993–2014, reaching 70% of samples [45]. People who consume a primarily organic diet excrete less glyphosate in their urine [46].

Impacts of agricultural practices, like genetic modification of crops and extensive pesticide use, on human health are difficult to assess, since they depend on factors that cannot be controlled or easily measured for human study populations. Therefore, Chhabra et al. [47] used *Drosophila* fruit flies to begin to experimentally assess the health impacts of organic (non-GMO, pesticide-free) food. *Drosophila* provides an excellent model for studying toxicology, especially the genetic mechanisms and gene X toxicant interactions [48]. Their natural diet is similar to humans’ (includes protein, carbohydrates, and fats) and can be easily controlled in the lab. They are easy and inexpensive to maintain, and have a short adult lifespan and high reproductive rate, allowing for large sample sizes. *Drosophila* genetic models for many human health concerns show that we share genetic and biochemical pathways. While there certainly are important limitations in applying results from *Drosophila* and other invertebrates and model species to human health applications [48], for example that they have different methods of detoxification and that all species can differ in sensitivity to some toxins, health issues studied in *Drosophila* and considered to be diet-related in humans include longevity [49], type 2 diabetes (insulin resistance; [50]), cardiac disease [51,52], immune function [53], autism [54], and neurodegenerative disease [55]. Pesticides can affect the same aspects of health; for example, exposure may be linked to gastrointestinal disorders, obesity, diabetes, heart disease, depression, autism, infertility, cancer, and Alzheimer’s disease, among others (see discussion). Many genetic tools used to study disease models in *Drosophila*, including mutant lines and expression-controlled lines, can provide important insights into the relationship between agricultural practices, including pesticide application, and a variety of diet-related diseases.

The purpose of this study is to assess how agricultural practices of food production affect the health of *D. melanogaster*. Specifically, we separate the effects of genetic modification from those of pesticide use. We assess whether the detriments often seen with use of GMO feed results directly from genetic modification, or from the increase in pesticides (glyphosate) typically found in and on genetically modified foods, by measuring mortality and sub-lethal behavioral effects similar to those identified in other animals of herbicide tolerant, genetically modified corn and of three formulations of Roundup^®^ and a non-glyphosate based herbicide on a non-target animal model, the fruit fly *Drosophila melanogaster*.

### Specific Hypotheses

We have tested two hypotheses that could explain previous analyses demonstrating harm from consuming genetically modified food and feed, each of which was tested by addressing several sub-hypotheses.

H_1_: Genetically modified, herbicide tolerant corn (NK603) harms non-target animals (*Drosophila melanogaster*) that eat it.

H_1-A_: Genetically modified corn increases mortality.
H_1-B_: Genetically modified corn decreases male courtship behavior.
H_1-C_: Genetically modified corn decreases mating behavior.
H_1-D_: The genetically modified corn used contains glyphosate residue.

H_2_: Glyphosate-based herbicide harms non-target animals (*Drosophila melanogaster*) when exposed through food.

H_2-A_: Exposure to glyphosate-based herbicide increases mortality.
H_2-Ai_: Mortality depends on herbicide formulation.
H_2-B_: Mortality depends on *D. melanogaster* genetic background.

## 2. Materials and Methods

### 2.1. Stock Maintenance

Flies of the Canton-S strain of *Drosophila melanogaster* were obtained from Erik Johnson and maintained in single-generation, mixed sex groups in 300 mL bottles containing approximately 50 mL of standard medium made with organic corn (Bob’s Red Mill) and molasses (Wholesome) and non-GMO, naturally sourced yeast and agar (Frontier). Bottles were maintained at 25 °C on a 12:12 light:dark cycle. Flies were transferred to new bottles without anesthesia, and old bottles discarded after approximately one month.

Flies for all experiments were collected within six hours of eclosion. They were transferred to an empty bottle and anesthetized with CO_2_, then transferred to a blue-ice pack to keep them asleep during sexing with the aid of a dissecting microscope, or were kept asleep during sexing using CO_2_ administered through a flypad (Genesee Scientific).

### 2.2. Experimental Set-up to Assess Genetically Modified Corn Toxicity

Three experiments were done to assess the lethal and sub-lethal effects of genetically modified corn, each using four treatments. Flies were exposed to standard medium as above, except made with either the same organic commercial corn used to maintain stocks (a corn that humans might be exposed to), or one of three types of corn grown by and obtained from a farmer in Iowa, USA. Herbicide tolerant w/ Roundup^®^ treatments contained genetically modified, herbicide tolerant corn, event NK603 (Pioneer 36V52 RR), which was sprayed with Roundup^®^ Weather Max at the rate of 32 oz/acre, twice during the growing season, at V3-4 and V7-8 stages of growth but not during the last three months before harvest. The herbicide tolerant unsprayed treatment was made from the same strain of NK603 (Pioneer 36V52 RR) genetically modified, herbicide tolerant corn grown without applications of Roundup^®^ during the growing season. Non-GMO unsprayed refers to a treatment that contains non-GMO corn from the near-isogenic, NK603 progenitor strain into which the herbicide tolerant construct had not been inserted (Pioneer 36V52). All NK603 and isogenic non-GMO corn was grown in Iowa in 2013 in the same location and conditions. The seeds used to grow the corn used in these treatments were pre-treated with Captan Fungicide (https://www.rrsi.com/wp-content/uploads/Captan_Fungicide_LABEL1.pdf); and the herbicides Callisto (1 oz./acre) and Steadfast (0.75 oz/acre), encapsulated in a soybean oil product that increases their efficiency, were also applied to all three Iowa plots. All fertilizers and other inputs were identical leading up to and during the 2013 growing season.

#### 2.2.1. Mortality

Our first experiment evaluated mortality of mature, adult flies, comparing those exposed to the four treatments beginning as three day old adults. Groups of ten male and ten female newly eclosed flies were transferred to 28.5 mm × 95 mm vials with organic commercial medium for three days. They were then anesthetized, separated into single-sexed groups, and transferred to vials containing one of the four treatments. Flies were transferred to new vials containing the same treatment, without anesthesia, every three days until all flies in the vial had died. Each vial was scored daily by counting the number of flies still alive, distinguishing between flies that died naturally and those who died of other causes, e.g., being trapped between the vial and stopper or escaping during transfer to a new vial. Seventeen replicate vials of each treatment were scored, with the exception of the herbicide tolerant unsprayed treatment, for which there were 15 replicates. In total, date of natural mortality was recorded for 137 female and 134 male flies exposed to the organic commercial treatment, 124 female and 138 male flies exposed to the non-GMO unsprayed treatment, 115 females and 112 males exposed to the Roundup^®^ ready unsprayed treatment, and 149 females and 129 males exposed to the Roundup^®^ ready w/Roundup^®^ treatment, resulting in a total of 1038 flies scored. The median age of death for each vial was used for statistical comparisons to avoid pseudo-replication. Average median survival data were compared using a multivariate analysis of variance using the identity matrix to test for significance. In addition, Cox proportional hazards risk ratios were used to analyze median survival across the lifespan, using diet, sex, and the interaction between the two as factors. Since the interaction term was significant, the analysis was repeated for males and females separately.

#### 2.2.2. Female Reproductive Behavior

The second experiment evaluated mating behavior in order to assess the toxic but sub-lethal effects of genetically modified corn on an important life-history trait strongly associated with female fitness. Flies were collected in single-sex groups of 10 and transferred to one of the four treatments for seven days. Without anesthesia, all males from a single treatment vial (average = 9.1 males) were transferred to an empty vial, followed by all females from a single vial of the same treatment (average = 9.1 females). The number of mating pairs in each group of flies was then scored once every five minutes for a total of 30 min, and the total number of pairs that mated during any part of the duration of observations recorded. Since successful mating usually lasts for at least 20 min [56,57,58], this protocol allowed every mating pair to be observed. The proportion of pairs mated in each replicate was calculated as:

$$\mathrm{Proportion}\;\mathrm{mated}=\frac{\#\;\mathrm{pairs}\;\mathrm{mated}}{\min\left(\#\;\mathrm{females},\;\#\;\mathrm{males}\right)},$$

where min(# females, # males) is the lesser of the number of males or the number of females in the vial after accounting for deaths and escapes. This was repeated for 24 sets of flies exposed to organic commercial corn medium, 23 sets exposed to non-GMO unsprayed, 25 sets exposed to Roundup^®^ ready unsprayed, and 23 sets exposed to Roundup^®^ ready w/ Roundup^®^, resulting in a total of 814 pairs of flies tested. Proportions mated for each treatment were compared using an analysis of variance.

#### 2.2.3. Male Reproductive Behavior

The third experiment evaluated male courtship behavior as an assay for sub-lethal toxicity. Since this is an energetically demanding and reproductively important behavior with strong implications for fitness, it should provide a good assessment of overall fly health, and male behavior is a relevant focus since males are sometimes more sensitive to dietary stress than females [59]. Unlike the previous assays, this one evaluated the effect of both larval and adult diet. Mixed sex groups of adult flies were transferred into bottles containing medium made with one of the four corn treatments, and allowed to mate and lay eggs. Newly eclosed flies from each of these bottles were collected and housed in single-sexed groups in vials of medium from each of the four corn treatments, creating a 4 × 4 larval diet × adult diet design, for a total of 16 treatment combinations. Observations of courtship behavior were made using a mating wheel [60] composed of four stacked plastic disks. The two inner disks each contained 10 holes, while the outer two disks contain a single, smaller hole offset from those in the inner disks. The solid parts of the outer disks form the top and bottom of chambers created out of the holes in the inner disks. With the holes in the two inner disks offset from each other, individual males were introduced into each chamber in one disk, and individual females from the same larval and adult diet treatment to chambers in the other disk. The disks were then gently rotated so the holes in the two inner disks line up, creating ten larger chambers each containing one male-female pair. Each pair was observed once per minute for 10 min, and courtship behaviors recorded as none, singing, following, attempted mating, or mating, and combinations of singing, following, and attempted mating. Rate of courtship was calculated for each pair as:

$$\mathrm{Rate}\;\mathrm{of}\;\mathrm{Courtship}=\frac{\sum \#\;\mathrm{courtship}\;\mathrm{behaviors}\;\mathrm{observed}}{\#\;\mathrm{observation}\;\mathrm{periods}\;\mathrm{preceding}\;\mathrm{mating}}.$$

A total of 152 pairs of flies were observed: For larval food treatments, 28 replicates of organic commercial, 39 of non-GMO unsprayed, 36 of Roundup^®^ ready unsprayed, and 49 of Roundup^®^ ready w/Roundup^®^ were observed; for adult food treatments, 34 replicates of organic commercial, 34 of non-GMO unsprayed, 36 of Roundup^®^ ready unsprayed, and 48 of Roundup^®^ ready w/Roundup^®^ were observed. Results were analyzed by multivariate analysis of variance using the identity matrix to test for significance. This was done in two different ways, first using “larval diet” and “adult diet” as the independent variables, and then using four independent variables: “Larval exposure to GMO corn”, “larval exposure to Roundup-treated corn”, “adult exposure to GMO corn”, and “adult exposure to Roundup-treated corn”.

### 2.3. Analysis of Glyphosate Residue on Corn

Three corn samples were tested for concentration of glyphosate and AMPA residue. These were organic commercial, herbicide tolerant w/Roundup^®^, and a commercially purchased yellow cornmeal grown conventionally and sold by Quaker Oats (conventional commercial). Samples were extracted and analyzed by Health Research Institute Laboratories, Inc. using high-performance liquid chromatography coupled with mass spectrometry. Effective concentration was calculated as [glyphosate] + 1.5* [AMPA] (Fagan, Heath Research Institute Laboratories, Inc., personal communication).

### 2.4. Experimental Set-Up for Herbicide Toxicity

Lethal effects of glyphosate-based herbicides were explored using three experiments that measured mortality of mature adult flies exposed to standard organic medium spiked with different concentrations of the appropriate herbicide. The first experiment assessed toxicity of a single herbicide at six concentrations; the second assessed genetic variation among wild-type flies in sensitivity to herbicide exposure; and the third compared the toxicity of three different herbicide formulations. In all three experiments, groups of ten male and ten female newly-eclosed flies were transferred to vials containing organic medium for seven days. They were then separated into single-sex groups and transferred to one of the relevant treatments for an additional seven days. Survival was scored after two days and seven days of exposure (i.e., nine and 14 days since eclosion). Relative survival was calculated as:

$$\mathrm{Relative}\;\mathrm{Survival}=\frac{\left(\frac{\#\;\mathrm{flies}\;\mathrm{alive}\;\mathrm{at}\;T_{i}\;\mathrm{in}\;\mathrm{the}\;\mathrm{treatment}}{\#\;\mathrm{flies}\;\mathrm{introduced}\;\mathrm{to}\;\mathrm{the}\;\mathrm{treatment}\;\mathrm{at}\;T_{0}}\right)}{\bar{x}\left(\frac{\#\;\mathrm{flies}\;\mathrm{alive}\;\mathrm{at}\;T_{i}\;\mathrm{in}\;\mathrm{the}\;\mathrm{matched}\;\mathrm{control}}{\#\;\mathrm{flies}\;\mathrm{introduced}\;\mathrm{to}\;\mathrm{the}\;\mathrm{matched}\;\mathrm{control}\;\mathrm{at}\;T_{0}}\right)},$$

where $T_{0}$ is the day flies were transferred to the herbicide treatments and $T_{i}$ is an observation made *i* days after the flies were transferred to treatments. The matched control contains flies of the same strain, sex, and age as the treatment, housed with organic medium without herbicide. Relative survival data were analyzed using JMP statistical software by multivariate analysis of variance (MANOVA) with repeated measures to account for scoring the same vials after two and seven days of exposure. In addition, regression analysis of relative survival on herbicide concentration was used to calculate concentrations at which 50% of the flies died (LC_50_) for each categorical treatment combination, using the equation for the line produced in the regression analysis; however, LC_50_ is only meaningful for those treatments in which survival of the matched control exceeds 60%, and the regression between relative survival and herbicide concentration is significant.

#### 2.4.1. Mortality

In the first herbicide mortality experiment, the lethal effects of Roundup^®^ Concentrate Plus were determined by measuring survival of flies exposed to one of five concentrations of glyphosate acid equivalent, 0.01 g/L, 0.1 g/L, 1 g/L, 2 g/L, and 10 g/L, plus the organic control. Roundup^®^ Concentrate Plus contains glyphosate and diquat as active ingredients and POEA as a surfactant. We replicated each treatment ten times, resulting in a total of 556 females and 563 males used in 120 experimental vials.

As a rough measure of reproductive success, the presence or absence of larvae was scored in each of the 60 vials of females after seven days of exposure to the treatments, and analyzed in JMP using a Pearson *χ^2^* Test.

#### 2.4.2. Genetic Background

The influence of the genetic background was assessed by comparing the sensitivity to Roundup^®^ of Canton-S flies with those of Harwick, both wild-type strains of *D. melanogaster*. The experimental treatments used our standard organic lab medium, with the addition of Roundup^®^ Super Concentrate at 0 (control), 2, 5, and 10 g/L. Roundup^®^ Super concentrate contains POEA as a surfactant in addition to the active ingredient glyphosate. In this experiment, survival was scored for males and females combined, and each strain by concentration combination was repeated 13 times resulting in a total of 52 vials of each strain scored (1034 Canton-S flies and 1026 Harwick flies). In the MANOVA, a significant interaction between strain and concentration would indicate an effect of the genetic background on sensitivity to Roundup^®^.

#### 2.4.3. Herbicide Formulation

Since other researchers have emphasized the importance of herbicide formulation (e.g., [61]), this experiment assessed mortality after exposure to herbicide formulations with different active ingredients. (1) Roundup^®^ Super Concentrate contains glyphosate as the only active ingredient, but also contains POEA (Liza Kronk, Consumer Response Representative, The Scotts Company and Subsidiaries, personal email 03/06/2017), a surfactant previously demonstrated to increase glyphosate toxicity [62]. (2) To compare to a formulation without the surfactant POEA (Carly Stidam, Consumer Response Representative, The Scotts Company and Subsidiaries, personal email 03/07/2017), we used Roundup^®^ Ready-to-Use, which contains both glyphosate and pelargonic acid as active ingredients. (3) Since another ongoing experiment in our lab indicated that Roundup^®^ Ready to Use might have stronger behavioral effects than Roundup^®^ Super Concentrate, we also utilized Scythe^®^, a pelargonic acid based herbicide that does not contain glyphosate. In all cases, other ingredients in the formulations are unknown because they are considered trade secrets and therefore proprietary information. Single-active-ingredient herbicides were used at 0.1, 1, and 10 g/L of the active ingredient (glyphosate or pelargonic acid), while Roundup^®^ Ready to Use was only used at two lower concentrations because the volume required to be added to medium to make the 10 g glyphosate/L treatment would dilute the nutrient composition of the food, creating additional variables. Since herbicide was always added after cooking, reducing the amount of water to compensate for herbicide volume was also not an option, as it would result in scalding. One treatment of this formulation contained 0.1 g/L of each glyphosate and pelargonic acid (a total of 0.2 g/L herbicide), while the other contained 1 g/L of each (2 g/L total herbicide). Each formulation by concentration by sex combination was replicated 12 times for a total of 216 vials scored. After scoring and statistical analysis, a significant interaction between formulation and concentration would indicate variability in toxicity of different formulations.

## 3. Results

### 3.1. Genetically Modified NK603 Corn Does Not Harm D. melanogaster’s Lifespan or Reproductive Behavior

**Figure 1:**
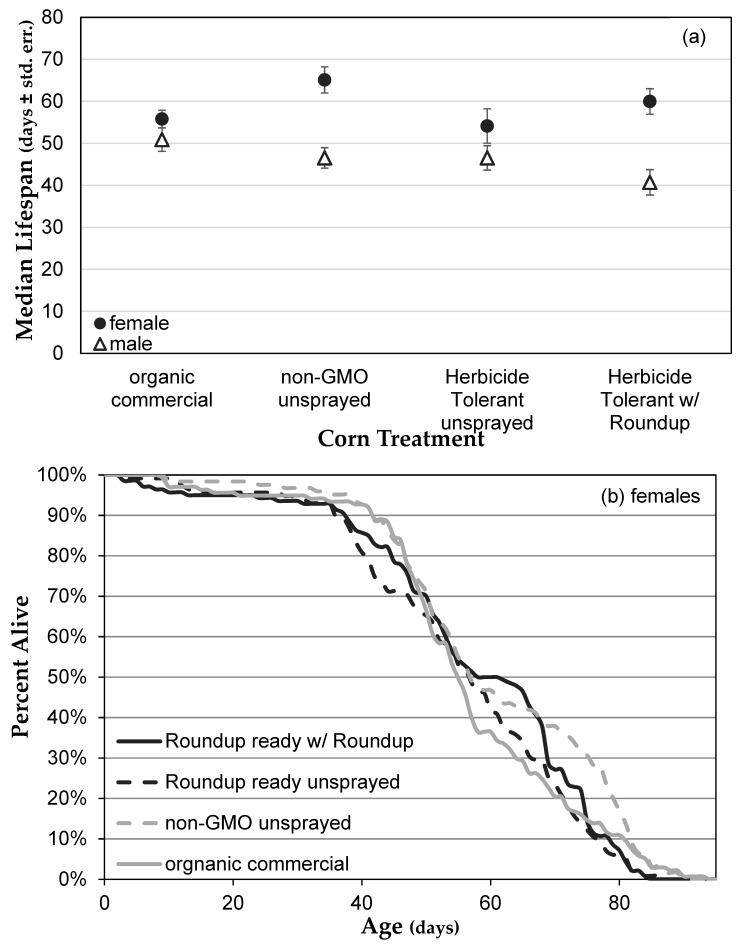

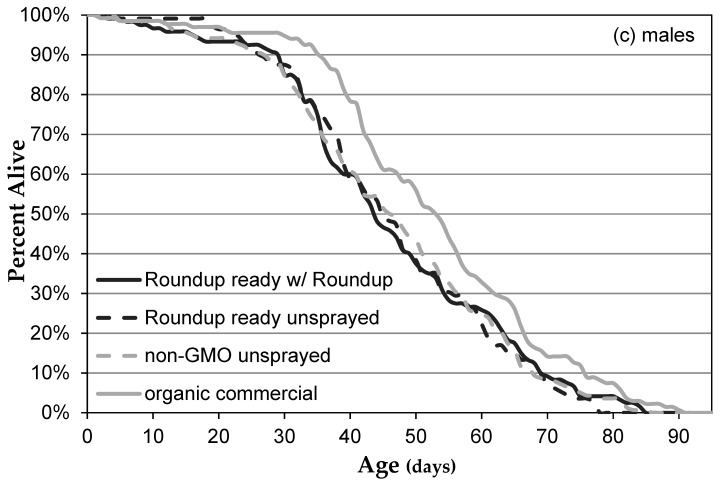
Lifespan of *Drosophila* exposed to diets containing different types of corn. (**a**) Lifespan (± standard error) of males (△) and females (**●**) for each diet treatment. (**b**,**c**) Time course of mortality for females (**b**) and males (**c**) on each diet treatment.

In the first experiment, we recorded the lifespan of males and females over the duration of their entire life. As expected, median male lifespan was shorter than that for females (factor sex, MANOVA: *F* = 35.86, *p* < 0.0001; Cox proportional hazards: Wald *χ*^2^ = 34.67, *p* < 0.0001; Figure 1a). However, lifespan was not affected by the type of corn in the diet overall (factor diet, MANOVA: *F* = 1.577, *p* = 0.1985; Cox proportional hazards: Wald *χ*^2^ =2.878, *p* = 0.411; Figure 1a). While it appears that for males, the organic treatment had longer lifespan than the other treatments, this difference was not significant as part of a whole model analysis (by sex = male, factor = diet, MANOVA: *F* = 2.292, *p* = 0.0874; Cox proportional hazards: *χ*^2^ = 7.313, *p* = 0.0626; Figure 1c). When all pairs of diets are analyzed by sex rather than including sex in the model, the difference between organic and Roundup^®^ Ready with Roundup^®^ becomes significant (Cox proportional hazards, risk ratio = 2.706, *p* = 0.0073), while other comparisons are not (remaining *p*-values range from 0.146 to 0.869). Using the same analyses overall (by sex = female, factor = diet, MANOVA: *F* = 2.428, *p* = 0.0739; Cox proportional hazards: *χ*^2^ = 7.023, *p* = 0.0712; Figure 1b) and with pairwise comparisons of the four diets for females, lifespan is slightly shorter for females exposed to conventional corn than to organic (risk ratio = 2.299, *p* = 0.0238) or Roundup^®^ Ready without Roundup^®^ (risk ratio = 2.164, *p* = 0.0416), while the other four comparisons are not (remaining *p*-values range from 0.098 to 0.866). However, with six pairs of comparisons, the appropriate *p*-value for comparison (Bonferroni correction) was *p* < 0.008, so that none of the comparisons for females were significant. Therefore, genetically modified corn in the diet did not alter lifespan in this experiment, while the presence of Roundup increased mortality for males only, and only when compared to the organic corn, not to Roundup^®^ Ready corn grown without Roundup^®^ application.

**Figure 2:**
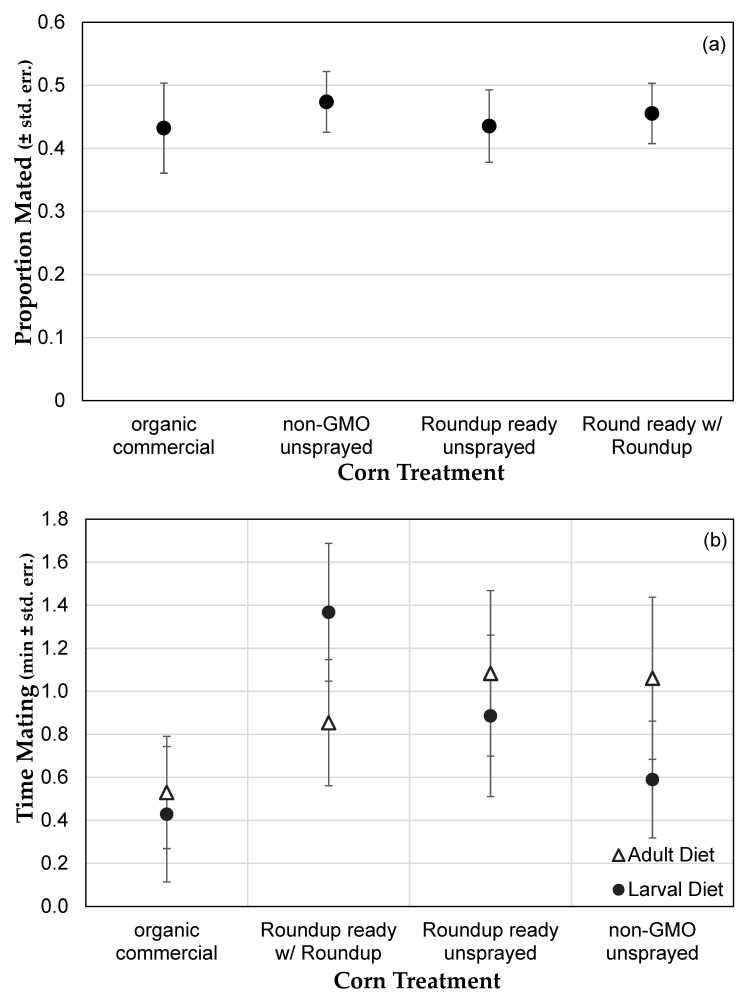

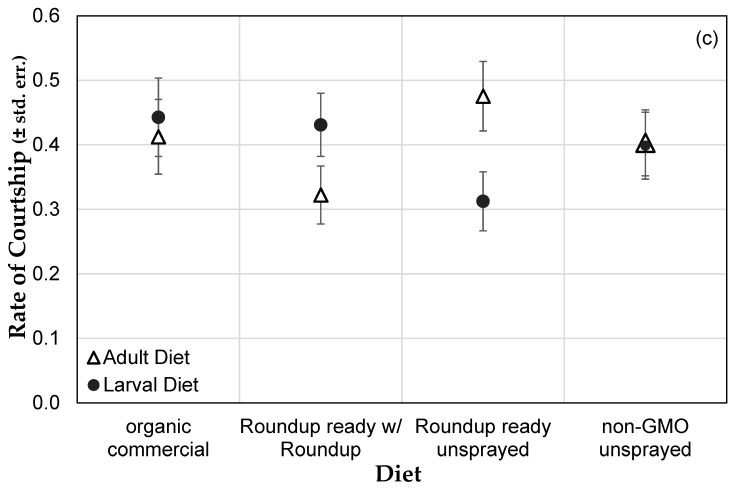
Effect of diets containing different types of corn on reproductive behavior. (**a**) Proportion of pairs mating (± std. err.), primarily controlled by females, in Experiment 2, of flies reared on different diets as adults. (**b**) Amount of time spent mating (± std. err.) in Experiment 3, which varied both larval (**●**) and adult (△) diets independently. (**c**) Amount of courtship by males (± std. err.) in Experiment 3 depending on larval (**●**) and adult (△) diets.

Neither female nor male reproductive behaviors were affected by genetically modified or Roundup^®^ treated corn in the diet. Mating propensity, usually controlled by females in *Drosophila*, especially when courtship vigor was taken into account, did not differ during the 30 min experiment 2 addressing adult diet only (*F* = 0.1354, *p* = 0.9387; Figure 2a), with an average of 44.7% of pairs mating. In the 10 min experiment 3, an average of 3.7 pairs mated in each trial out of an average of 8.6 pairs present in the vial (43.0%), but was independent of both larval diet (*F* = 1.052, *p* = 0.3717) and adult diet (*F* = 0.3144, *p* = 0.815; Figure 2b). Even the one non-significant difference in mating behavior in experiment 3 is in the opposite direction of that expected (flies exposed to organic food mated less than those in other treatments), and was not consistent with the results of experiment 2. In addition, male courtship vigor, measured in experiment 3, was consistent across larval diets (*F* = 1.022, *p* = 0.3851) and adult diets (*F* = 1.098, *p* = 0.3522; Figure 2c). There was a nearly-significant trend that males exposed to corn treated with Roundup as adults, but not as larvae, court less vigorously than those not exposed (MANOVA, *F* = 3.904, *p* = 0.0501). This indicates that these dietary treatments affect neither female nor male reproductive behavior, though adult exposure to Roundup at this concentration may have a slight effect on male courtship behavior only.

### 3.2. The NK603 Genetically Modified Corn Used Contains Low Concentration Glyphosate Residue

Chemical analyses of the corn samples indicated that only very low concentrations of glyphosate residue were present in any of the corn samples (Table 1). Roundup^®^ Ready corn grown with Roundup^®^ had the highest effective concentration of the three, 8.05 ng/g of corn. As used to make medium according to the recipe we used in these experiments, that would have resulted in 4.4 × 10^−7^g glyphosate/L medium, more than seven orders of magnitude less than the LC_50_ for males (see Section 3.2). Our negative control, Bob’s Red Mill Organic Corn Meal, contained no detectable glyphosate or AMPA residue. Conventional commercial corn was not used in any experiments with flies, but was tested for comparison to corn to which humans might be likely to be exposed.

### 3.3. Herbicide Exposure Increases Drosophila melanogaster Mortality

Exposure to diets containing added Roundup^®^ increased female and male fly mortality in a dose-dependent manner for nearly all herbicide formulations tested, regardless of the flies’ genetic background, after two days or seven days of exposure or both. The significant and meaningful LC_50_ values range from 2.67 g/L for female Canton-S flies exposed to Roundup^®^ Concentrate Plus for seven days to 11.5 g/L for female Canton-S flies exposed to Roundup^®^ Concentrate Plus for two days (Table 2). The mean of all meaningful and significant LC_50_s across formulations, sexes, strains, and exposure times was 9.16 g/L. Since it is unknown how much each fly eats, we were unable to convert this into a lethal dose (LD_50_).

#### 3.3.1. Roundup^®^ Exposure Causes a Dose-Dependent Mortality Increase

**Figure 3:**
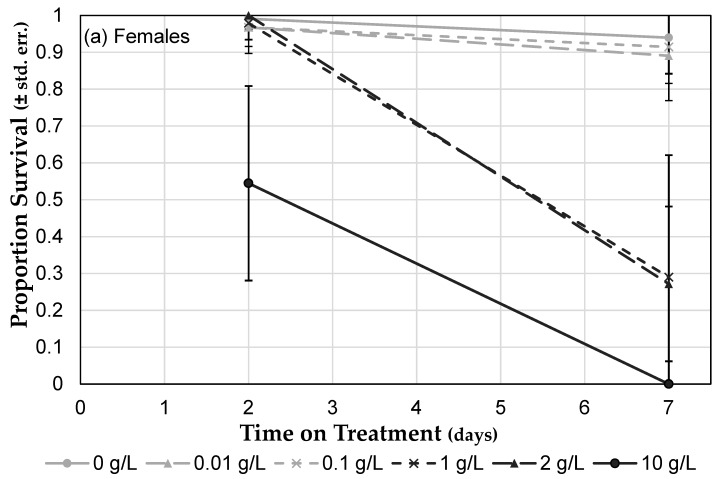

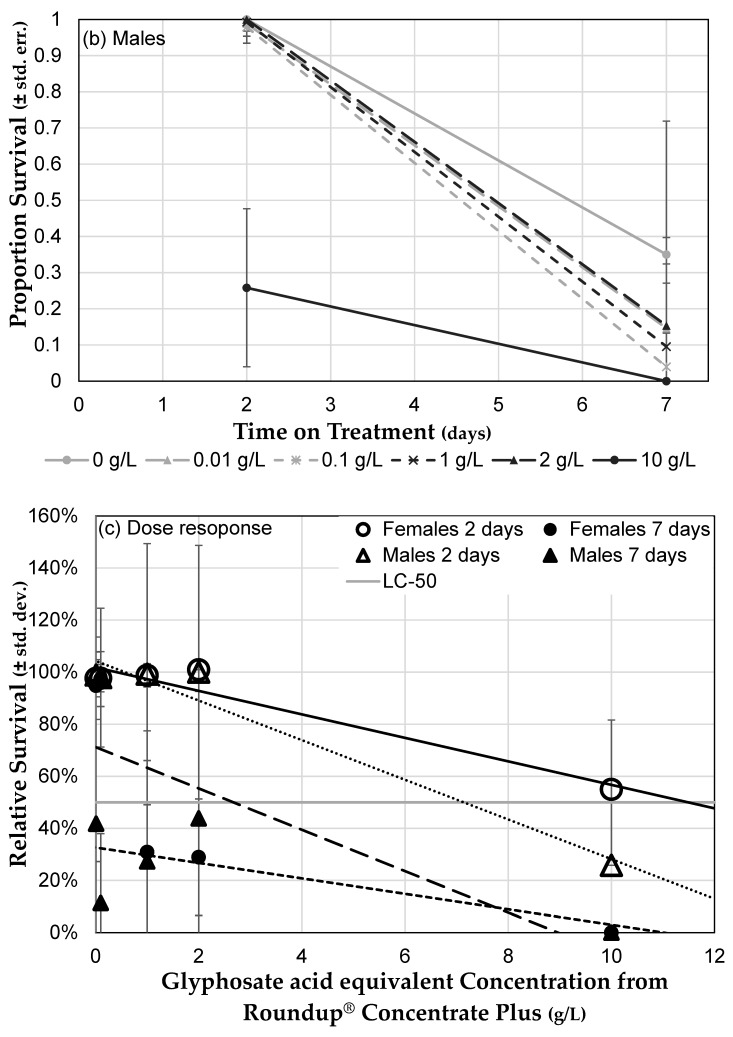
Roundup^®^ exposure in diet influences *Drosophila* mortality. (**a**) Female mortality (± std. err.) after two days and seven days of Roundup^®^ exposure (nine days and 14 days post eclosion) at six concentrations. (**b**) Male mortality (± std. err.) after two days and seven days of Roundup^®^ exposure. (**c**) Survival of females and males after two days and seven days of Roundup^®^ exposure compared to the survival of flies exposed to control medium (± std. dev.). The light grey line indicates 50% survival and can be used to determine LC_50_ for two-days of exposure.

Survival depended on concentration of Roundup^®^ Concentrate Plus (treatment: *F* = 242.44, *p* < 0.0001), differed by sex (sex: *F* = 83.97, *p* < 0.0001), and affected females and males differently (sex × treatment interaction: *F* = 6.05, *p* = 0.0154). For females, only 10 g/L glyphosate was significantly different from the no-Roundup^®^ control after two days of exposure, but by seven days of exposure 1 g/L glyphosate and above decreased survival (Figure 3a). Males exhibited the same dose response after two days of Roundup^®^ exposure; but by seven days, survival of all treatments, including the control, had decreased to less than 40%, and those trials exposed to 10 g/L suffered 100% mortality (Figure 3b). Glyphosate concentration correlated negatively with survival for both females and males at both two and seven days of exposure (Table 2). Based on the equation for regression lines, using data from two days of exposure, LC_50_ was calculated to be 11.4 g/L for females and 7.1 g/L for males (Figure 3c).

**Figure 4:**
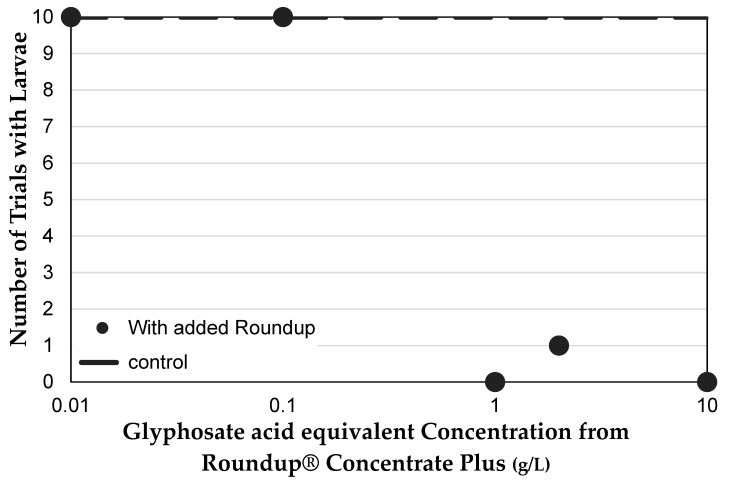
Effect of Roundup^®^ and glyphosate concentration on reproduction. Number of trials in which vials contained larvae after adult females had been present from day 7 to day 14 after eclosion. Lack of larvae most likely indicates high mortality of eggs (before or after they are laid) or early instar larvae.

In addition to adult mortality, the presence of larvae was scored for each female vial (*n* = 10 for each Roundup^®^ concentration). All no-Roundup^®^ control vials contained larvae, as did those containing 0.01 g/L and 0.1 g/L glyphosate. However, of the thirty vials that contained medium with 1 g/L, 2 g/L or 10 g/L glyphosate, only one contained any larvae (*R*^2^ = 0.9218, Pearson *χ*^2^ = 56.396, *p* < 0.0001, Figure 4). Since adult females were not exposed to Roundup^®^ until after they were seven days post eclosion, it is unlikely that ovary development or mating behavior account for this effect. In addition, average mortality was low after two days of exposure in all female trials, so adult females were present to lay eggs. Therefore, this most likely indicates that Roundup^®^ exposure increases mortality of eggs or early instar larvae.

#### 3.3.2. Genetic Background Does Not Influence Sensitivity to Herbicide

**Figure 5:**
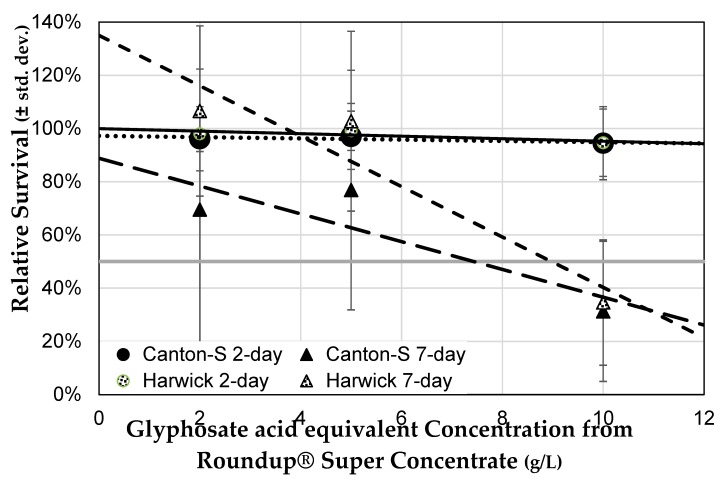
Effect of the genetic background on sensitivity to Roundup^®^ concentration. Survival of mixed-sex groups after two days and seven days of Roundup^®^ exposure (nine days and 14 days post eclosion) at three concentrations of Roundup^®^ Super Concentrate in food medium, compared to the survival of flies of the same age and strain exposed to control medium (± std. dev.). The light grey line indicates 50% survival and can be used to determine LC_50_ for seven-days of exposure.

Flies from the two wild-type strains of *Drosophila* exhibited a similar response of mortality when exposed to Roundup^®^ Super Concentrate in their food medium (strain: *F* = 5.43, *p* = 0.0225; concentration: *F* = 25.4, *p* < 0.0001; strain × concentration: *F* = 2.15, *p* = 0.1467; Figure 5). However, the two strains differed slightly in their response to duration of exposure (exposure: *F* = 3.01, *p* = 0.0871; exposure × strain: *F* = 7.18, *p* = 0.0091; exposure × concentration: *F* = 35.7, *p* < 0.0001). In both strains, the response was non-significant after two days of exposure, with nearly as many exposed flies surviving all treatments as those exposed to control medium. However, after seven days both Canton-S and Harwick flies showed a similar dose-dependent decrease in survival (Table 2). The only difference was that at low herbicide concentration, the relative survival of Canton-S flies was lower than of Harwick flies. While other strains would need to be examined, this may suggest that there is little variation among wild-type laboratory strains of *Drosophila* in their sensitivity to glyphosate-based herbicides.

#### 3.3.3. Herbicide Formulations Differ in Toxicity

**Figure 6:**
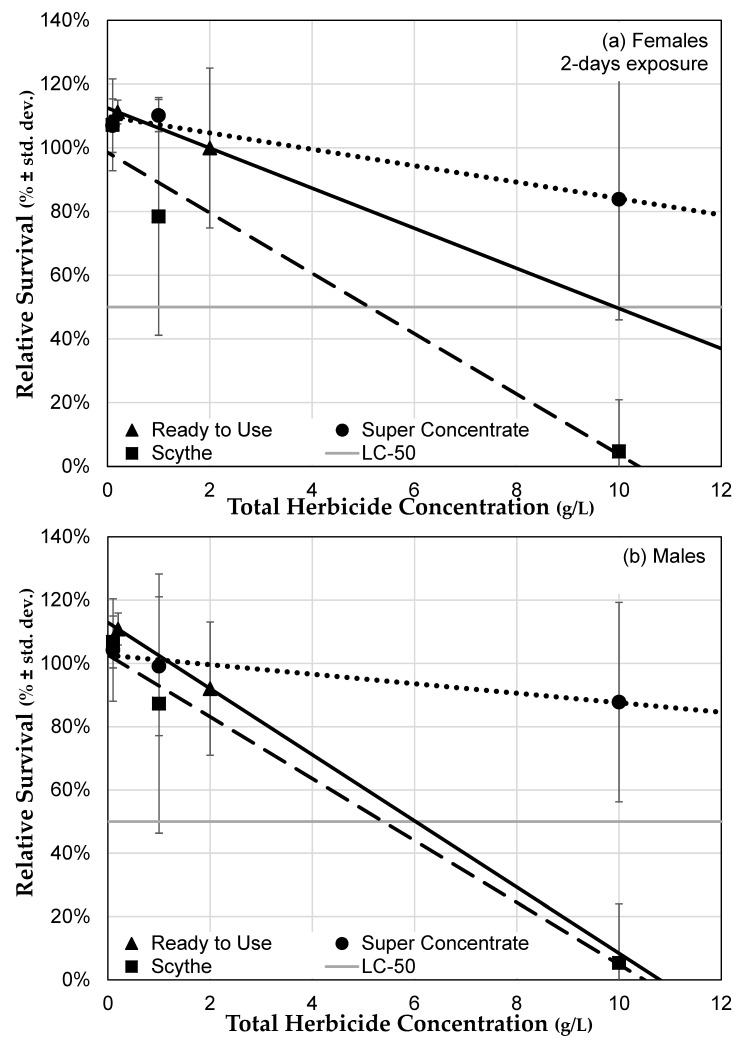
Effect comparison of the effects of three herbicide formulations on relative survival. Survival of (**a**) females and (**b**) males after two days of herbicide exposure (nine days post eclosion) to three herbicide formulations in food medium, compared to the survival of flies of the same age and sex exposed to control medium (± std. dev.). The light grey line indicates 50% survival and can be used to determine LC_50_ for some formulations.

Herbicide formulation does affect toxicity to *D. melanogaster* flies (MANOVA: Formulation *F* = 3.455, *p* = 0.0338; sex *F* = 4.864, *p* = 0.0287; herbicide concentration *F* = 46.40, *p* < 0.0001; formulation × sex *F* = 1.910, *p* = 0.1513; formulation × concentration *F* = 6.007, *p* = 0.0030; sex × concentration *F* = 5.873, *p* = 0.0164; formulation × sex × concentration *F* = 1.873, *p* = 0.1568; Figure 6). Among those formulations that we compared directly, herbicides containing pelargonic acid tend to be more toxic than those that contain glyphosate as the only active ingredient. We were unable to determine whether Roundup^®^ Ready to Use, which contains both glyphosate and pelargonic acid herbicides, was more similar to a glyphosate-only formulation of Roundup^®^ or to Scythe^®^, which contains only pelargonic acid as an active ingredient, since we were unable to test it at all the concentrations since the formulation itself was more dilute. However, Scythe^®^ certainly increased mortality at lower concentrations than glyphosate-based Roundup^®^ Super Concentrate, suggesting that a pelargonic acid based herbicide might be even more toxic. For example, the LC_50_ for seven-day old females exposed to Roundup^®^ Super Concentrate was 5.43, while for the same type of flies exposed to Scythe^®^ the LC_50_ was 3.04. Similar patterns occurred for two-day old females and for males at two and seven days, though not all regressions remained significant after Bonferroni correction (Table 2).

## 4. Discussion

The lifespan of flies from the two control corn treatments (organic commercial and non-GMO unsprayed) in Experiment 1 of this study (females 61 days, males 49 days) was moderately consistent with other studies (ex. unmated females 47 days, mated females 35 days, males 34 days, [63]; females 44–53 days, males 54–59 days, [64]; females 18–47 days, males 18–42 days, [65]). The only difference in lifespan seen was between males and females. Males are expected to have shorter lifespans (ex. [65]), since selective pressures for extended lifespan are constrained by factors associated with seeking and obtaining mates [66] and estrogen upregulates some longevity-associated genes [67].

Our results showed no significant differences in mortality between flies grown on medium made with organic corn and those made with non-GMO unsprayed corn, Roundup^®^ ready unsprayed corn, or Roundup^®^ ready corn sprayed with Roundup^®^. We also found no significant difference in the proportion of flies that courted and mated when reared on the different corn-diet treatments. All flies mated in relatively consistent proportions, suggesting that eating genetically modified corn did not affect the reproductive behavior of male or female flies. A difference in lifespan, mating, and/or courtship behavior between the organic commercial and non-GMO unsprayed control treatments, and the Round-up Ready^®^ sprayed and unsprayed treatments would have indicated that genetic modification itself affected fly health. Since there were no differences among these groups of treatments, our results suggest that this particular event of the genetic modification of corn with the Roundup^®^ Ready construct (NK603) did not influence mortality or reproductive behavior of animals eating the modified corn. While we could make the more general conclusion that genetic modification does not cause lethal or overt sub-lethal affects, several critically important limitations must be considered. First, the trait inserted must be considered. We know of no a priori reason to expect the ESPS protein itself to harm either the crops into which it is inserted or organisms eating those crops. Yet the same may not be true of some other genes used to produce agriculturally important GMO seeds, such as the various genes that code for insecticides in the insect tolerant lines of corn and cotton that are very commonly used in industrial agriculture. Second, consuming genetically modified food may influence more subtle aspects of health that we did not measure, including protein expression, behavior, morphology, physiology, or histology, or in ways that only become apparent in some circumstances, for example under stressful conditions (ex. oxidative stress response [68]; by increasing response to food allergens, [69]). Third, each event of genetic modification must be considered independently, even if the same cassette is inserted into the same crop, since transformation can result in genome-wide off-target mutations (reviewed in [70,71]). For example, it may depend on where the insertion lands: How it affects nearby genes, promoters, enhancers, and other regulatory elements, whether it disrupts protein folding sequences, and whether it interferes with DNA replication. Transcriptome and proteome analyses have demonstrated that genetic manipulation causes unintended molecular differences related to altered protein expression in crop plants [33,72,73,74,75,76]. Organisms eating these GM crops may also have altered transcriptomes [34]. Even if changes in transcription, translation and protein modification are limited to the crop itself, they may cause or increase the expression of allergenic proteins in the food [77]. Importantly, all of these effects are likely to vary depending on where in the genome the transgenes insert, which will differ in each transformed cell created. This study and those cited above have used commercially available strains of genetically modified feed, but more modern methods of genetic modification, like the CRISPR-Cas9 system, also create unintended, off-target genetic changes [78,79,80,81].

We particularly expected to see a difference in lifespan and behavior between flies grown on organic corn medium and those grown on herbicide tolerant corn sprayed with Roundup^®^, since glyphosate has been shown to increase mortality in *Drosophila melanogaster* [82,83]. However, since our Roundup^®^ ready corn sprayed with Roundup^®^ contained very little glyphosate residue (see Table 1), this treatment did not affect lifespan or behavior; however, further behavioral studies are needed to assess the effects of higher concentrations of glyphosate-based herbicides on reproductive behavior and development. Studies in other species indicate that Roundup^®^ and glyphosate exposure do result in behavioral consequences among some invertebrates (ex. earthworm avoidance and feeding [84], *Daphnia* juvenile behavior [85], honeybee flight [86] and feeding behavior [87], beetle locomotor speed and avoidance response [88], orb weaving spider web building, prey consumption and egg laying [89], and wolf spider mate localization [90]) and vertebrates (ex. frog tadpole antipredator behavior [91], rat depressive behavior [92], and mouse anxiety and depression [93]).

Flies exposed to known concentrations of glyphosate from Roundup^®^ in their medium did experience high mortality. Our results indicated that the LC_50_ for glyphosate in Roundup^®^ Concentrate Plus of fully mature adult male flies after two days of exposure was 7.1 g/L, was 11.4 g/L for females, and was lower after seven days of exposure. These concentrations generally agree with previous results for *Drosophila* [82,83], and are fairly similar to the LC_50_ found for honeybees in direct contact with glyphosate for 24 h (LC_50_ = 5.11 g/L for *Apis mellifera* and 5.09 g/L for *Hypotrigona ruspolii*; our calculations based on data in [94]). Concentrations that increased mortality are well above those found on the corn samples we tested (Section 3.2) and used in the experiments reported above (Section 3.1). However, they are releant both because other foods, including soybeans [42] and those containing oats [41], contain higher concentrations of glyphosate residue and because flies and humans exposed during application would experience a higher concentration. For example, one of the formulations we used, Roundup Ready-to-Use, is intended to be used at full-strength, yet we diluted it from an initial concentration of 12 g/L glyphosate acid equivalent into *Drosophila* food to a final concentration of 1 g glyphosate/L. Therefore, both the concentrations that we tested and the calculated LC_50_s are in the environmentally relevant range.

While we did not quantify food consumption, making direct comparisons to other kinds of feeding trials impossible, we estimated that this concentration in the food would result in flies consuming in the range of 200–500 g glyphosate/kg body weight/day (Elias et al., unpublished). This is much higher than the lethal doses in rats treated intratracheally (0.2 g glyphosate/kg, [95]) and earthworms in soil (<20 mg/kg, [96]; <50 g/kg, [97]). In fact, a review of the effects of glyphosate and Roundup^®^ below regulatory limits found that teratogenic, tumorigenic, and hepatorenal effects regularly occur below the official lowest observed adverse effect level [61]. Most other studies reporting lethal concentrations are in aquatic organisms, and report the concentration in the water, which is not comparable to quantity ingested.

In this study, sensitivity of *Drosophila* to glyphosate-based herbicide was not affected by the genetic background of wild-type flies, suggesting little genetic variation for this trait among different lab populations; but did vary by herbicide formulation, with formulations containing pelargonic acid, with or without glyphosate, among the most toxic. Since a number of studies indicate that other ingredients found in some glyphosate based herbicide formulations, particularly POEA, do increase the toxicity of glyphosate, for example [34,61,62,98,99], we directly compared a formulation with POEA, Roundup^®^ Super Concentrate, and without POEA, Roundup^®^ Ready to Use. Unfortunately, the later also contains the herbicide pelargonic acid, likely added to consumer ready-to-use formulations because it increases the short-term visual injury to plants, even though it has little effect on long-term weed control [100]; or because it increases the penetration of other ingredients [101]. The effects of glyphosate and pelargonic acid may be additive, since the slope of survival after exposure to Roundup^®^ Ready to Use is intermediate between Roundup^®^ Super Concentrate and Scythe^®^ when total herbicide concentration ([glyphosate] + [pelargonic acid]) is considered. Few previous studies have examined the toxicity of pelargonic acid to animals. One exception is the work of Techer et al. [102,103], who demonstrated lethal and sub lethal toxicity to zebrafish, including LC_50_ = 81.2 mg/L, and decreased hormone levels and liver damage at sub lethal concentrations. Our results corroborate the importance of formulation in elucidating herbicide toxicity.

Consistent with reproductive effects of glyphosate seen in other animals, a nearly complete decrease in the presence of larvae was seen in vials of flies fed medium containing Roundup^®^ Concentrate Plus with glyphosate concentrations of 1 g/L of glyphosate or higher, while all other treatments and controls contained many larvae. Glyphosate-based herbicides alter concentrations of reproductive hormones, for example testosterone in rodents [104,105,106,107], estradiol in drakes, *Anas platyrhynchos*, [108] and rats [109], and prolactin in rats [105]. Physiologically, glyphosate based herbicides decrease sperm concentration and sperm count [104,105,110], induce earlier onset of male [106], and cause apoptosis in testicular cells [107] in rats; impair spermatogenesis, decrease sperm motility and concentration, increase sperm deformity, and induce germ cell apoptosis in male mice [111]; and alter testes and epididymal structure of drakes [108]. Human sperm motility and mitochondrial metabolism are decreased by Roundup^®^ exposure [112]. In females, glyphosate decreases fecundity and fertility and affects ovary and oocyte development in the spider, *Alpaida veniliae* [89] and the terrestrial snail, *Cantareus aspersus* [113]; and disrupts uterine development [114], decreases the number of implantation sites [115], and increases latency to the first mount [109] in rats. Developmentally, maternal glyphosate exposure of rats delays fetal growth and induces structural congenital anomalies [115], and slows growth and development of the aquatic larval stages in common toads [116].

Other studies demonstrate non-reproductive, sub-lethal health effects of glyphosate or Roundup^®^ exposure (reviewed in [117,118]). In humans, for example, those who are chronically diseased have more glyphosate residue in their urine than healthy people [46]. Roundup^®^ disrupted several measures of endocrine function in human liver cell lines [98], breast cancer cell lines [119], cholangiocarcinoma cell lines [120], and placental cell lines [99], though the role of estrogen receptors (ERα) has been debated [119,120,121]. Glyphosate was listed by the IARC, a branch of the World Health Organization, as a class 2a “probable human carcinogen” [122], based on evidence from animal models and because the herbicide has been implicated in human non-Hodgkin lymphoma [123,124]. The mechanism of carcinogenicity may be DNA damage that results from the increase in reactive oxygen species induced by Roundup^®^ and its ingredients, as shown to occur in human blood cells [125] and *Drosophila* [83]. Behavioral (autism, seizure disorder: [126,127], digestive [128], and metabolic complications may be related to Roundup^®^-induced changes in the gut microbiome (humans: [129,130]; poultry: [131]; cattle: [132,133]; mice: [93]; and rats: [134]). Damaged kidney function seen in agricultural workers was attributed to exposure to Roundup^®^ along with heavy metals in the drinking water [135]. Finally, acute ingestion of Roundup^®^ at very high doses, for example during suicide attempts, leads to death because of cardiac failure [136,137].

In addition to modifying the composition of gut microbe communities, glyphosate changes the composition of soil [138] and rhizome-associated microbial communities [139,140]. Since glyphosate can persist in the soil for decades, depending on the soil type [141], this can result in long-term degradation of soil as it has in Argentina [142]. Glyphosate also bioaccumulates within soil-building and plant-decomposing organisms, particularly when applied in commercial formulations [143]. If glyphosate-induced soil contamination, microbiome alteration, and macrobiotic bioaccumulation become widespread, it could decrease agricultural productivity in areas with particular soil compositions.

While our results do not indicate that NK603 herbicide tolerant corn severely affects *Drosophila melanogaster* health in the absence of herbicide exposure, our results are consistent with other studies that indicate that Roundup^®^ exposure does. A few other studies have distinguished between unsprayed herbicide tolerant genetic modification and Roundup^®^ exposure. For example, Malatesta et al. examined hepatocytes from mice fed GMO soybeans [21], and from tissue culture exposed to low concentrations of Roundup^®^ [22], finding similar cellular aberrations and dysfunctions, but did not include a treatment fed GMO soybeans grown without Roundup^®^ application. Séralini et al. [9] exposed rats to both genetically modified corn with and without Roundup^®^ application and to Roundup^®^ alone, finding that similar pathologies result from consumption of GMO corn, whether sprayed or unsprayed, and from Roundup^®^ alone. Perhaps the most complete analysis that directly compares the effects of a Roundup-Ready crop to the effects of Roundup itself is the work of Bøhn, Cuhra and others using genetically modified soybeans fed to *Daphnia magna*. Mortality of *Daphnia* was affected by both glyphosate and Roundup in the water, as was behavioral, developmental, and reproductive processes [144]. They then quantified the glyphosate residue load and nutritional characteristics of 31 soybean samples that were Roundup-Ready, non-GMO conventional or organic [42], and used these to identify that life-cycle differences occur among these categories [145], and among the GMO soy samples, correlate with glyphosate residue concentration [146]. While a GMO unsprayed treatment was not included, the quantification that the least severe affects occurred when glyphosate residue was lowest indicates that the presence of glyphosate is likely to be more important than the genetic modification *per se*, in this system as well as in *Drosophila*.

## 5. Conclusions

Lifespan, dietary behavior, and reproductive behavior were uninfluenced by dietary exposure to herbicide tolerant corn of the strain NK603 in the fruit fly *Drosophila melanogaster*. Conversely, dietary exposure to all three formulations of glyphosate-based herbicides tested impacted mortality and reproduction at concentrations as low as 1 g/L. While this suggests that herbicide exposure is a greater health risk than exposure to genetically modified foods, we caution that this conclusion should be limited to this particular strain (NK603) of this particular modification (glyphosate resistant). In addition, pelargonic acid-based herbicide increased *Drosophila* mortality, both as the only active ingredient and in a formulation containing glyphosate as well. Our results confirm some of the harmful effects of glyphosate-based herbicides that have been shown for other non-target organisms, and establish *Drosophila* as a useful tool to explore the genetic and biochemical mechanisms responsible.

## Figures and Tables

**Table 1:** Glyphosate analysis for three types of corn.

Treatment	Glyphosate (ng/g)	AMPA (ng/g)	Effective Concentration in Corn (ng/g)	Effective Concentration in Medium (μg/L)
Organic commercial	Not detected	Not detected	Not detected	Not detected
Roundup^®^ Ready w/Roundup^®^	5.67	1.52	8.05	0.437
Conventional commercial	0.42	0.21	0.74	Not used in medium

**Table 2:** Relative survival and herbicide concentration. Regression analysis of survival of *D. melanogaster* exposed to herbicide in their food medium, as a proportion of survival of flies exposed to organic control medium, on herbicide concentration for different durations of exposure, sexes, herbicide formulations, and wild-type strains of *Drosophila*. Significant *p*-values shown in bold. LC_50_ is not meaningful if regression *p* is NS. ^a^ LC_50_ is not meaningful because the mortality rate for controls was >50%.

Experiment	Formulation (Active Ingredient) (other known ingredients)	Strain	Sex	Exposure	R^2^	F Ratio	*p*-value	LC-50 (g/L)
Exp. 1 Bonferoni-corrected *p* = 0.0125	Roundup Concentrate Plus (glyphosate, diquat) (POEA)	Canton-S	Females	2 days 7 days	63.4% 47.6%	83.3 43.5	<0.0001 <0.0001	11.5 2.67
			Males	2 days 7 days	86.2% 5.6%	300 2.84	<0.0001 0.0985	7.12 −5.86^a^
Exp. 2 Bonferoni-corrected *p* = 0.0125	Roundup Super Concentrate (glyphosate) (POEA)	Canton-S	Both	2 days 7 days	0.40% 14.2%	0.150 6.11^t^	0.7007 0.0181	199 7.43
		Harwick	Both	2 days 7 days	2.9% 50.6%	1.10 37.9	0.3008 <0.0001	106^a^ 8.97
Exp. 3 Bonferoni-corrected *p* = 0.0042	Roundup Super Concentrate (glyphosate) (POEA)	Canton-S	Females	2 days 7 days	21.6% 92.1%	9.11 397	0.0049 <0.0001	23.3 5.43
			Males	2 days 7 days	8.0% 39.6%	2.87 22.3	0.0999 <0.0001	34.9 6.51^a^
	Roundup Ready to Use (glyphosate, pelargonic acid)	Canton-S	Females	2 days 7 days	9.4% 29.7%	2.19 9.29	0.1534 0.0059	9.93 3.12
			Males	2 days 7 days	29.3% 37.8%	8.30 13.4	0.0092 0.0014	6.02 3.41^a^
	Scythe (pelargonic acid)	Canton-S	Females	2 days 7 days	75.1% 53.4%	99.3 38.9	<0.0001 <0.0001	5.14 3.02
			Males	2 days 7 days	75.7% 36.2%	103 19.3	<0.0001 <0.0001	5.40 7.47^a^
